# Covid‐19 laryngectomized patients care, on field experience, and considerations

**DOI:** 10.1002/ccr3.3953

**Published:** 2021-02-21

**Authors:** Filippo Ricciardiello, Michele Caraglia, Gian Marco Romano, Giuseppe Longo, Giuseppe Russo, Salvatore Mazzone, Nunzio Accardo, Teresa Abate, Flavia Oliva, Giovanni Motta, Marco Bocchetti

**Affiliations:** ^1^ Ear, Nose, and Throat Unit AORN "Antonio Cardarelli" Naples Italy; ^2^ Department of Precision Medicine University of Campania "Luigi Vanvitelli" Naples Italy; ^3^ Biogem Scarl Molecular Oncology and Precision Medicine Laboratory Ariano Irpino Italy; ^4^ UOC Post‐operative Therapy AORN "Antonio Cardarelli" Naples Italy; ^5^ Healthcare Direction AORN "Antonio Cardarelli" Naples Italy; ^6^ General Direction AORN "Antonio Cardarelli" Naples Italy

**Keywords:** COVID‐19, infectious diseases, laryngectomy, SARS‐CoV‐2

## Abstract

Laryngectomized patients showed an unconventional response to SARS‐CoV‐2 viral infection. Here, we describe five different patient cases along with our interpretation of the phenomena and suggestions for their safe management.

## Graphical abstract

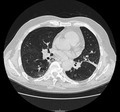

## 1 INTRODUCTION

COVID‐19 pandemic is now relapsing worldwide, it is important to control the diffusion and, at the same time, improve patients' conditions. Therefore, it is crucial to establish strong protocols and rules to follow in order to treat specific and different subsets of patients, in our case Total laryngectomized patients.

SARS‐CoV‐2 (Coronavirus 2) is spreading worldwide causing a severe acute respiratory syndrome: COVID‐19. The first sight of the virus was reported in Hubei, China province, but nowadays it has become pandemic.^2^ To date, COVID‐19 caused over 46.000.000 confirmed cases worldwide and over 1.000.000 deaths.^3^

COVID‐19 symptomatology is enormously variable, ranging from asymptomatic or mild condition to severe illness and death. Common symptoms include cough, fever, and shortness of breath. Other reported symptoms are weakness, malaise, respiratory distress, muscle pain, sore throat, loss of taste and/or smell^4^ and also dyspnea and radiological signs. Although severe lung injury has been described at all ages, the virus is more likely to cause severe interstitial pneumonia, acute respiratory distress syndrome (ARDS), and subsequent multiorgan failure into elderly or patients affected with multiple comorbidities. This frequently results in severe acute respiratory failure and high death rates in this fragile subset of patients.^5^, ^6^

Reliable diagnostic COVID‐19 testing is crucial for healthcare institutions during the critical pandemic condition we are now facing, to appropriately treat the positive patients and eliminate the risk of disease spreading to other negative patients. Gold standard for COVID‐19 diagnosis is reverse transcription polymerase chain reaction (RT‐PCR) aiming to detect SARS‐CoV‐2 specific genes from respiratory tract origin samples. Test sensitivity is highly correlated with specimen type and collection technique but also with symptom onset. Specimen from lower respiratory tract, such as broncho‐alveolar lavage fluid, has a higher sensibility. Sputum, nasal swabs, and pharyngeal swabs have lower decrescent sensitivity.^7^

Total laryngectomy (TL) is performed in patients showing larynx and pharynx advanced malignancy, but, nowadays, the indication for TL is restricted and applied less frequently. This is mainly due to the development and increased efficacy of less drastic techniques like partial laryngectomy and radiotherapy. However, TL remains an intervention practiced in a conspicuous number of patients, for example, during 2013 in the US, 3389 patients undergone this intervention.^1^, ^8^ In a total laryngectomy, the lungs and the lower trachea are completely disconnected from nose, mouth, and pharynx, with the airflow starting directly through a tracheostoma above suprasternal notch; the patient is unable to emit sound and the upper respiratory tract is inactive.

The COVID‐19 TL patients' management requires special consideration for the peculiar postsurgical anatomy, the report of five cases gave us the opportunity for interesting considerations on COVID‐19 diagnosis and management in this particular subset of patients.

## 2 CASES REPORTS

### 2.1 Case 1

Male patient, 71 years old, 77 kg (BMI: 23), smoker (half pack a day).

High risk for unfavorable COVID‐19 prognosis due to previously diagnosticated comorbidities: virus related liver disease, Chronic Obstructive Pulmonary Disease (FEV 73%) and previous acute myocardial infarction in treatment with ACE‐inhibitor, cardio aspirin, and statin.

Moreover, in November 2017 he was diagnosed with glottic‐subglottic laryngeal carcinoma, pT3N2bM0, with subsequent total laryngectomy, bilateral neck dissection and adjuvant radiotherapy. The follow‐up did not evidence relapses, last TC total body in May 2020.

In September 2020, the patient was showing fatigue, arthralgia, airways mucositis, cough. Nasopharyngeal/Oropharyngeal swab and tracheal stoma swab resulted both positive for SARS‐CoV‐2, while blood exam was showing lymphopenia.

Fever (38.5°C) was only present for 1 day and medical therapy was conducted at home with Azithromycin 500 mg 1 pill/die for 6 days, Prednisone 25 mg 2 pills/die for 3 days and 1 pill/die for subsequent 2 days, Enoxaparin 6000 UI 1 fl sc/die and Paracetamol 1000 mg when needed. Oxygen therapy was not required and HRCT was not practiced. After 8 days, patient was not showing any COVID related symptom and after 15 days from diagnosis the molecular swab resulted negative.

All cohabiting became positive for COVID‐19.

### 2.2 Case 2

Male patient, 76 years old.

High risk for unfavorable COVID‐19 prognosis due to previously diagnosticated comorbidities: Chronic Obstructive Pulmonary Disease with frequent acute exacerbations, hearth failure by myocardial ischemia, high blood pressure in treatment with antiplatelets, diuretics, antiarrhythmics, and statin. In May 2016, he was diagnosed for laryngeal carcinoma, pT4N2bM0, with subsequent total laryngectomy, bilateral neck dissection and adjuvant radiotherapy. The follow‐up didn't evidence relapses, last TC total body in August 2020.

In October 2020, the patient presented fever, arthralgia, airways mucositis, and cough.

Nasopharyngeal/Oropharyngeal swab and tracheal stoma swab resulted both positive for SARS‐CoV‐2, while blood exam was not evidencing any lymphopenia. He was then admitted in COVID‐19 Cardarelli Hospital (Naples – Italy) department and the therapy consisted in Azithromycin 500 mg 1fl/die, Piperacillin/Tazobactam 4 + 0.5 mg 1 fl/three times per day, Dexamethasone, Mucolytics, Enoxaparin 6000 UI 1 fl sc/die. Oxygen therapy with low‐flow oxygen T‐tube was required and HRCT showed bilateral interstitial pneumonia (Score < 12^9^). After 10 days, patient was showing gradual amelioration in terms of COVID‐19 symptoms and after 15 days from diagnosis the molecular swab resulted negative.

Three cohabiting became positive for COVID‐19.

### 2.3 Case 3

Male patient, 54 years old, smoker (one and half pack a day).

Previous trauma with bone lesions and spleen‐nephrectomy, no cardiac disease. During October 2020, the patient was diagnosed with glottic laryngeal carcinoma, pT4aN2bM0, laryngectomy, and bilateral neck dissection were required. Postoperative course was regular with good results for the swallow test after 5 days.

**FIGURE 1:**
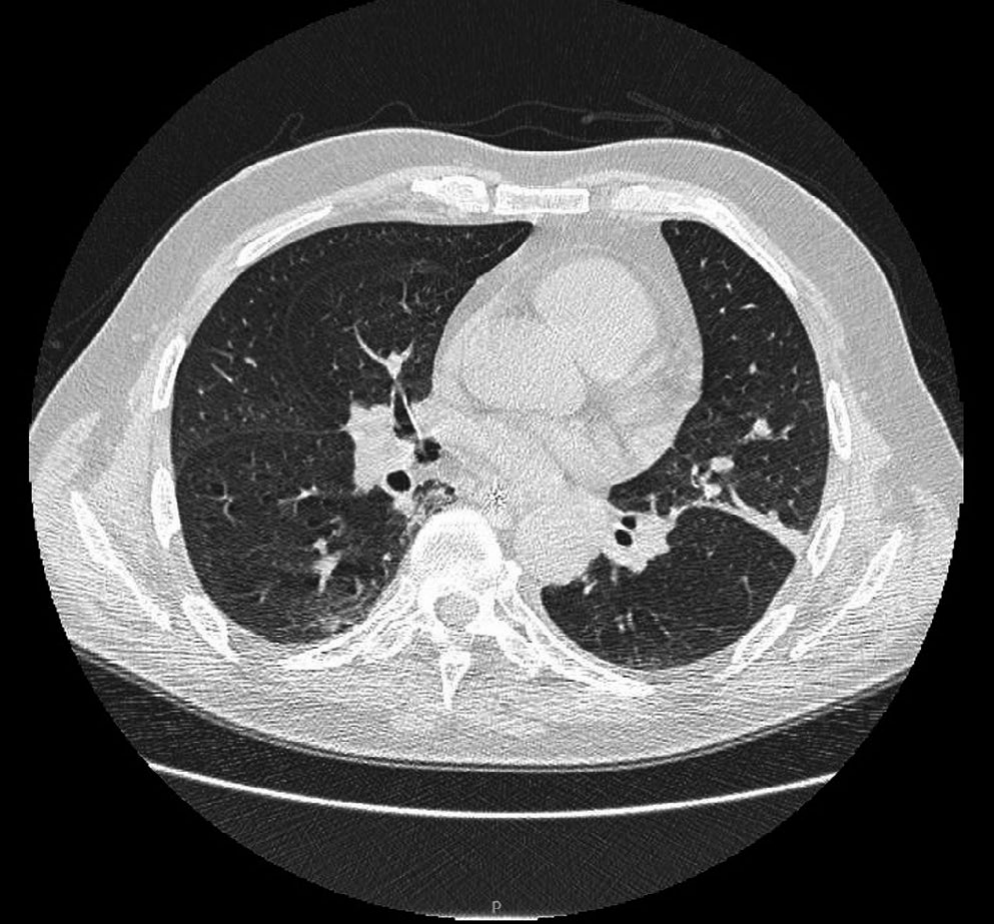
Thin layer of parietal basal pleural effusion on the left with minimal scissural effort

During the 7th day following the intervention, patient showed fever, fatigue, and arthralgia. Nasopharyngeal/Oropharyngeal swab and tracheal stoma swab resulted both positive for SARS‐CoV‐2, while blood exam was evidencing lymphopenia and hypoalbuminemia, moreover, SARS‐CoV2 related IgM presence and IgG absence were confirming the recent contamination. During the 8th day following the intervention, pharyngocutaneus fistula was forming probably due an infection favored by concomitant lymphopenia. Therefore, patient stopped oral feeding and started external nutrition. Medical therapy consisted in Azithromycin 500 mg 1fl/die, Piperacillin/Tazobactam 4 + 0.5 mg 1 fl/three times a day, Dexamethasone, Mucolytics, Enoxaparin 6000 UI fl sc/die. Low‐flow oxygen therapy through T‐Tube was required and HRCT showed a low score (Figure 1).

Three nurses were infected despite the use of PPE.

### 2.4 Case 4

Male patient, 81 years old, smoker (one pack per day).

High risk for unfavorable COVID‐19 prognosis due to previously diagnosticated comorbidities: Chronic Obstructive Pulmonary Disease and ischemic heart disease. In 2018, patient was diagnosed with glottis laryngeal cancer with subglottic extension, CT2N0M0, treated with radiotherapy. Total laryngectomy was required after recurrence in July 2020, follow‐up was relapse free.

In October 2020, patient showed fatigue, Nasopharyngeal/Oropharyngeal swab, and tracheal stoma swab resulted both positive for SARS‐CoV‐2, while blood exam was not evidencing any lymphopenia. COVID‐19 course was regular and symptoms resolved after 10 days from diagnosis. Twenty days after, diagnosis swabs resulted negative.

### 2.5 Case 5

Female patient, 62 years old.

High risk for unfavorable COVID‐19 prognosis due to previously diagnosticated comorbidities: virus‐related liver disease, esophageal varices, diabetes. Total laryngectomy was performed during 2002.

**FIGURE 2:**
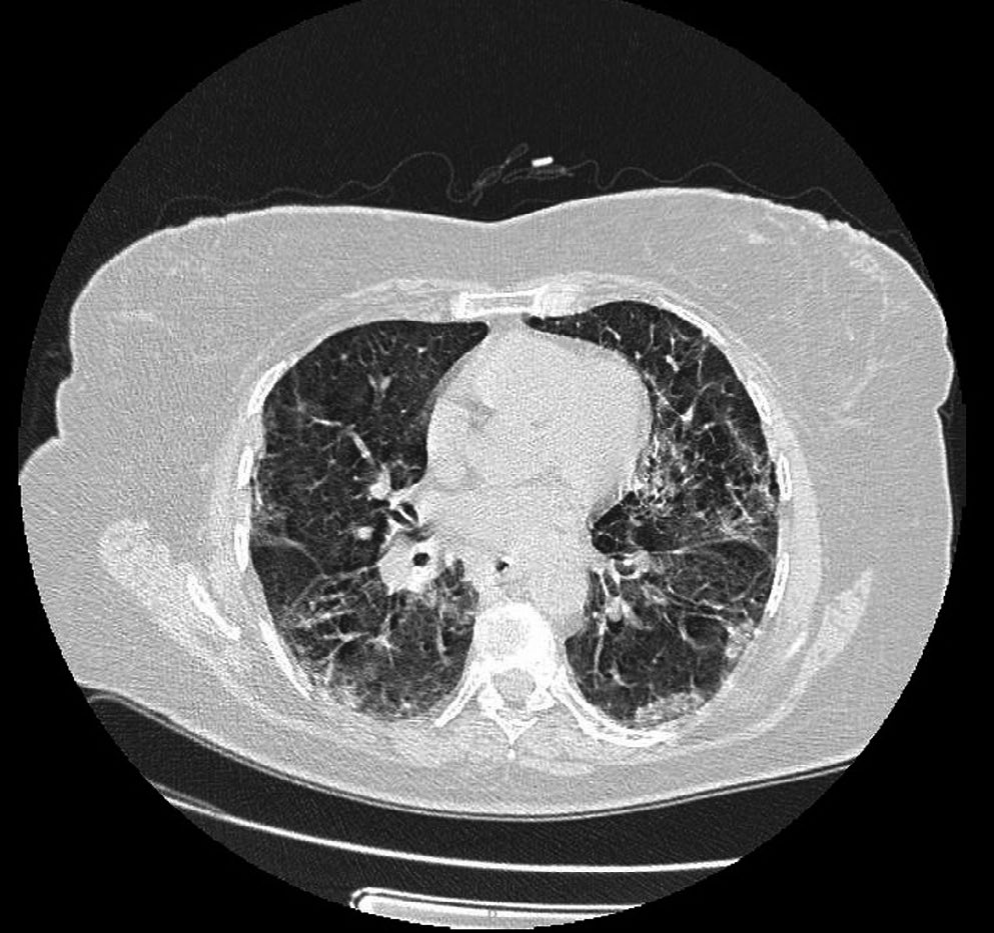
Presence of consolidation areas of the bilaterally ground glass type

In October 2020, patient showed fever and cough. Nasopharyngeal/Oropharyngeal swab and tracheal stoma swab resulted both positive for SARS‐CoV‐2, while blood exam evidenced lymphopenia. She was then admitted in COVID‐19 Cardarelli Hospital (Naples – Italy) department and the therapy consisted in Azithromycin 500 mg 1fl/die, Dexamethasone, Enoxaparin 6000 UI 1 fl sc/die. Oxigen therapy with low‐flow oxygen T‐tube was required and HRCT (Figure 2) had a score of 8.^9^ COVID‐19 course was regular with symptoms resolution and relocation to a secondary hospital.

Several family members became positive for COVID‐19.

## 3 DISCUSSION

In our patients, both nasopharyngeal and tracheal specimens tested positive for SARS‐CoV‐2. The virus is primarily transmitted via inhalation of respiratory droplets.^10^ In the TL patients, as we mentioned, the upper respiratory tract is inactive, therefore it logically follows that specimens obtained from their nasopharynx are unlikely to contain SARS‐CoV‐2. This conclusion is incorrect for two reasons: COVID‐19 pathophysiology and its mode of contagion. The Coronaviruses infect a wide variety of host species^11^ and several studies demonstrated that virus lifecycle within the host consists of the following 5 steps: attachment, penetration, biosynthesis, maturation, and release. Once viral particles bind to host receptors (attachment), they enter host cells through endocytosis or membrane fusion (penetration). Once viral contents are released inside the host cells, viral RNA enters the nucleus for replication. Viral mRNA is used to make viral proteins (biosynthesis) and then new viral particles are assembled (maturation) and released. Angiotensin‐converting enzyme 2 (ACE2) was identified as a functional required receptor for SARS‐CoV and its expression is high in nasopharynx, oropharynx, trachea, lungs, heart, ileum, kidney, cornea, and esophagus.^12^ Therefore, once its life cycle is complete, virus spreads to all tissues presenting the ACE2 receptor, especially in nasopharynx and trachea (even in laryngectomized patients). The second reason for upper airways and tracheostoma contagion in TL patients might be the contact with a contaminated surface such as own hands.^7^ The presence of tracheoesophageal prothesis (TEP) and its related fistula, connecting trachea, and esophagus, is important to be taken in account since infection to trachea might spread to the pharynx and vice versa. This is why both Nasopharyngeal/Oropharyngeal and tracheal stoma swabs remain crucial for diagnosis.^13^, ^14^, ^15^

Total laryngectomy patients are considered “super spreaders”, in fact they can generate a greater infected aerosol through the tracheostoma, and this was confirmed by our patients experience since they showed a remarkably higher capability to spread COVID‐19 infection to nearby family members, but also to healthcare operators wearing PPE, compared to nonTL individuals. Therefore, Max Hennessy et al recommend the use of enhanced PPE to assist those patients, and we completely agree. In particular, to minimize aerosolized particle spread, the tracheostoma should be covered with a heat moisture exchanger (HME), preferably with an integrated viral/bacterial hydroscopic filter. Important is a physical barrier over the stoma as well, such as a surgical mask, scarf, or shirt.^16^

Our cases presented previous cancer risk, radiotherapy, heart disease, and COPD so all of them were at very high risk for unfavorable COVID‐19 prognosis. Surprisingly, all of them resulted COVID‐19 paucisymptomatic: five out of five showed fatigue and asthenia, four out of five presented fever. Two out of five referred anosmia (even if olfaction is weaker in TL patients) and only two out of five showed pulmonary symptoms but easily resolved. Coleman et al ipotized that the lack of direct physical route from the nasopharynx to the lungs may result in a reduction of lung viral load, causing only mild illness in an otherwise well recognized high‐risk group.^17^ We think that COVID‐19 pathophysiology partially contradicts this opinion because when the viral life cycle is complete it still spreads to all tissues expressing ACE2 receptor into the host, upper airways included. On the other hand, is important to take into account that the time frame and viral load in the lungs derived from infection concomitant or starting from upper airways might be, respectively, faster and heavier compared to other noncontiguous tissues derived infection. This sort of “protection” might be the reason why TL high risk patients showed non‐critical COVID‐19 symptoms.

## 4 CONCLUSIONS

Our experience evidenced that the best COVID‐19 testing approach in laryngectomy patients remains the multiple‐site testing, meaning that the specimens should be collected from both the upper and aerodigestive track (NP swab) and lower respiratory track (tracheal swab).

Moreover, our cases also confirmed the capability of TL patients to be “super spreaders”, so their management specifically requires enhanced PPE for family and healthcare operators, cover of the tracheostoma with a heart moisture exchanger (HME), preferably with an integrated viral/bacterial hydroscopic filter, in addition to face masks is advised, as well.

Further study on a greater laryngectomy patient cohort is required to better clarify this complex and specific relationship with COVID‐19.

## CONFLICT OF INTEREST

The authors declare no conflict of interest.

## AUTHOR CONTRIBUTIONS

FR: involved in conceptualization, experimental design, and patient analysis; GMR, GL, GR, SR, NA, FO, TA, and GM: involved in supervision and patient analysis; MC and MB: involved in revision and manuscript writing and formatting.

## ETHICAL APPROVAL

The study was in accordance with the Institutional Ethics Committee guidelines, Italian law, and the Declaration of Helsinki. All patients provided informed consent regarding the use of these data for research purposes.

## Data Availability

Data sharing not applicable to this article as no datasets were generated or analyzed during the current study.
